# A Case of Incidentally-diagnosed Erdheim-Chester Disease

**DOI:** 10.7759/cureus.781

**Published:** 2016-09-13

**Authors:** Atman A Dave, Susan E Gutschow, Christopher M Walker

**Affiliations:** 1 Medical Education, Saint Luke’s Hospital of Kansas City; 2 Department of Radiology, Saint Luke’s Hospital of Kansas City

**Keywords:** erdheim-chester disease

## Abstract

Erdheim-Chester disease (ECD) is a rare multisystemic non-Langerhans cell histiocytosis that may be clonal and inflammatory in origin. The hallmark of the disease is infiltration of various organ systems by CD68+/CD1a- histiocytes containing foamy lipid-laden inclusions. The manifestations and course of the disease are variable and depend on the organ systems that are affected. Patients may be asymptomatic or may develop life-threatening complications, including myocardial infarction. The most common clinical manifestation is lower extremity bone pain. Imaging manifestations of the disease include symmetric osteosclerosis of the distal long bones, circumferentially “coated” aorta, pleural and pericardial thickening/fluid, and perirenal encasement. Treatment for the disease is evolving, particularly with the use of molecular BRAF inhibition. We present a case of a patient with ECD initially suspected based on the imaging manifestations.

## Introduction

Erdheim-Chester disease (ECD) is a rare multisystemic non-Langerhans cell histiocytosis first described in 1930 by Jakob Erdheim and William Chester. The disease is characterized by infiltration of skeletal, cardiac, and other major organ systems by histiocytes with lipid-laden cytoplasmic inclusions. To date, there have been less than 600 cases reported in the medical literature and little has been elucidated about the exact pathogenesis of the disease [1]. Patients may present with life-threatening complications of the disease, particularly compression of vital structures, or may be completely asymptomatic. ECD is progressive, and as such, incidental diagnosis and intervention can dramatically alter the course of the disease. Here, we present a case of a patient diagnosed with ECD initially suspected by radiologic findings.

## Case presentation

**Figure 1:**
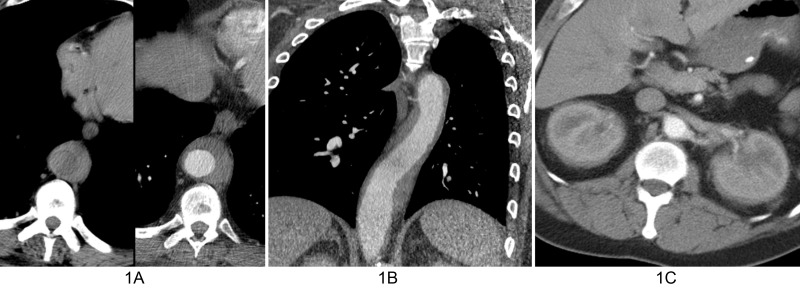
Computed tomography (CT) findings in of patient with Erdheim-Chester disease A. Composite image with axial precontrast (left image) and axial contrast-enhanced (right image) CT shows enhancing soft tissue (arrows) partially surrounding the descending thoracic aorta. B. Oblique coronal contrast-enhanced CT better shows the extent of peri-aortic soft tissue spanning the entire descending thoracic aorta. C. Axial contrast-enhanced abdominal CT shows symmetric perirenal infiltration by soft tissue (arrows).

**Figure 2:**
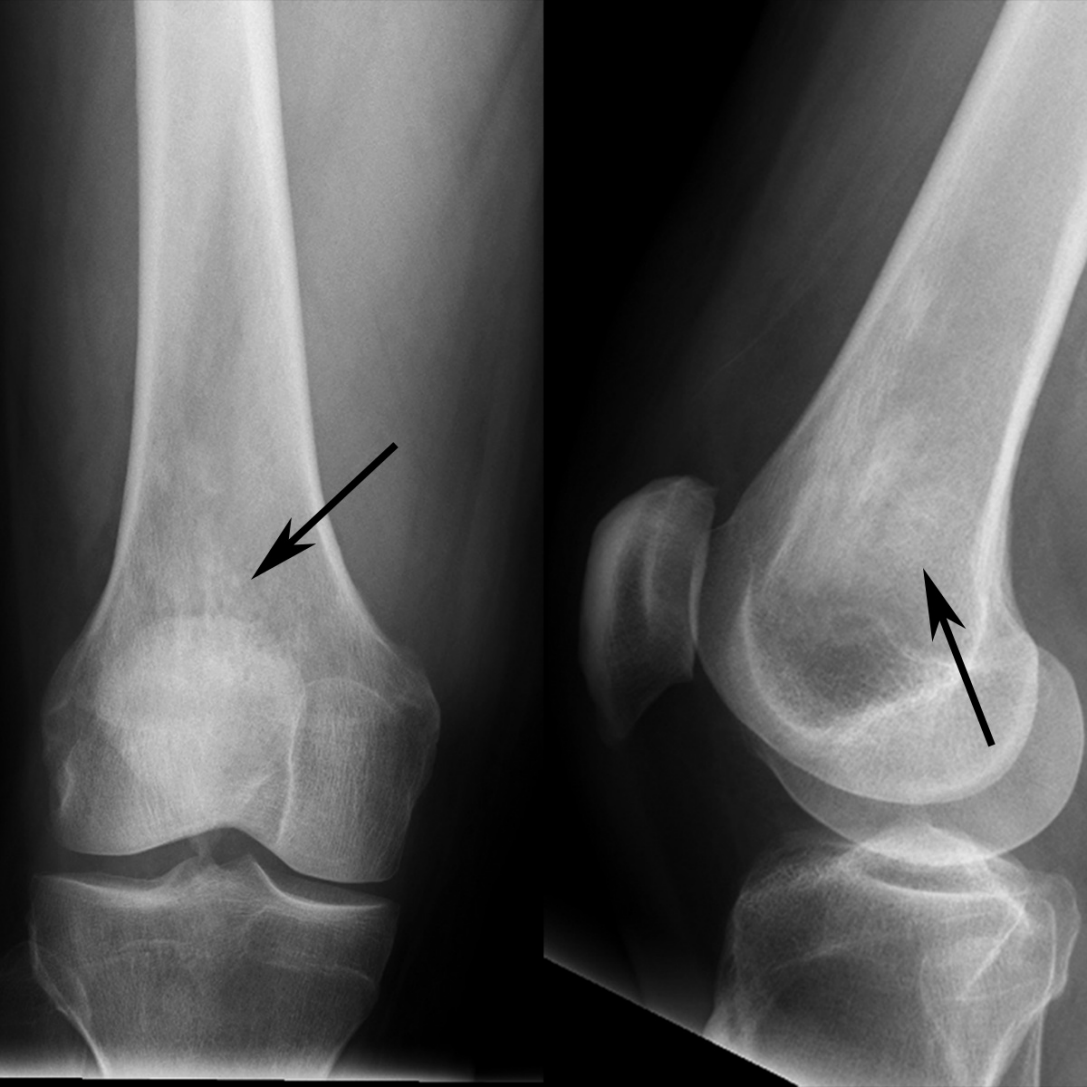
Femoral radiographs of patient with Erdheim-Chester disease Composite image of the distal femur with frontal radiograph (left image) and lateral radiograph (right image) shows patchy sclerosis (arrows) in the distal femoral metadiaphysis. The contralateral femur (not shown) had similar findings.

A 45-year-old man presented with dizziness and pain after sustaining trauma to his right upper back from the lid of his car. The patient’s past medical history was significant for myocardial infarction treated with multivessel coronary artery stenting. Physical examination revealed extensive swelling and hematoma of the right scapular region with extension into the lower back and right flank. No other abnormal physical exam findings were noted. Computed tomography (CT) of the chest and abdomen were performed and revealed a right chest wall periscapular intramuscular hematoma correlating with the recent injury. CT was also significant for diffuse, slightly irregular thoracic aortic wall thickening and perinephric soft tissue with a fullness of both renal collecting systems (Figure 1). Given the renal and vascular findings, a diagnosis of ECD was suspected. Bilateral femoral radiographs were obtained for confirmation and showed patchy osteosclerosis of the distal meta-diaphyseal regions of both femurs (Figure 2). Informed patient consent was obtained for this patient's treatment.

Outpatient testing confirmed the presence of a BRAF-V600E mutation identified on cfDNA testing. Histopathologic examination of a biopsy from the patient’s distal left femur demonstrated patchy bone marrow fibrosis associated with a variably cellular lymphocytic and foamy histiocytic infiltrate, with immunohistochemical studies positive for CD68 and negative for CD1a. The patient is currently in the process of enrolling in a clinical trial involving dabrafenib and trametinib therapy.

## Discussion

The clinical and imaging manifestations of ECD are diverse and can involve multiple organ systems. The pathophysiology of the disease is unclear, but ECD is thought to be a non-neoplastic pro-inflammatory disorder, though the recent discovery of the oncogenic BRAF-V600E mutation in patients has shifted this view to characterizing the disease as a clonal disorder with a characteristic inflammatory cytokine profile [2]. Histological demonstration of CD68+ and CD1a- foamy histiocytes is required for diagnosis in correlation with the appropriate clinical and imaging findings.

Clinically, ECD affects patients at a mean age of 53, with a slight male predominance [3]. Patients most frequently present with bone pain, and less commonly, exophthalmos, diabetes insipidus, fever, and weight loss [3]. Up to 96% of patients have skeletal involvement, and the majority of patients have osteosclerotic lesions, with a minority also having lytic lesions [4].

Cardiovascular manifestations of ECD include pericardial effusion, tamponade, congestive heart failure, valvular disorders, renovascular hypertension, periaortic fibrosis, and myocardial infarction, as was the case in our patient’s history [5]. In a review of 72 cases of ECD, Haroche, et al. demonstrated that 56% of patients had circumferential aortic encasement, the so-called “coated aorta” [6]. Of these 40 cases, 20 involved the entire aorta, 10 were limited to the thoracic aorta, and 10 were limited to the abdominal aorta. Aortic involvement in ECD may be distinguished from aortic involvement in Takayasu arteritis and retroperitoneal fibrosis (RPF) based on the pattern of infiltration and organ association. In ECD, fibrosis is largely found in the adventitia of the vessel wall. In contrast, Takayasu arteritis involves all layers of the vessel wall; however, this distinction will be difficult to appreciate on imaging. RPF involves the abdominal aorta, typically below the renal artery origins, and may extend to also affect the ureters and inferior vena cava [4-6].

Other organ systems commonly involved are the central nervous system and pulmonary system. Diabetes insipidus comprises about a fifth of initial manifestations of ECD due to compression or a lesion of the infundibular stalk [7]. Meningeal, parenchymal, and orbital involvement is also not uncommon and may manifest with dural masses, diffuse pachymeningeal thickening, enhancing intra-axial masses, or enhancing orbital masses. ECD may also affect the lung and pleura, with more than a third of patients having pulmonary involvement in one study [7]. Lung involvement often mimics interstitial pulmonary edema or lymphangitic carcinomatosis with interlobular septal thickening, peribronchovascular thickening, fissural thickening, and foci of ground-glass opacity [4, 8]. Pleural and pericardial effusions and/or thickening, as well as centrilobular nodules, are often present.

Our patient demonstrated a perinephric halo of soft tissue on abdominal CT, classically described as a “hairy” kidney. Renal visualization is enhanced by contrast and allows for differentiation of infiltrative tissue from the kidney [4]. Extensive fibrosis of the retroperitoneal space can be seen and may manifest as ureteral narrowing, though pelvic ureters are often spared [1-2]. This renal involvement may cause hydronephrosis or renal failure. The major imaging differential diagnosis for circumferential soft tissue encasing the kidneys is lymphoma.

Treatment and management of ECD are evolving as more is understood about the disease. No randomized control trials have been performed, and various modalities of therapy have been utilized, including interferon-alpha, BRAF inhibition, chemotherapy, steroids, and radiotherapy. Current guidelines encourage participation in a clinical trial, with thorough imaging evaluation and follow-up shown in Table 1 [2]. The therapy with the most evidence is interferon alpha, though reports of BRAF inhibitors providing a dramatic response in life-threatening cases have been recently reported [9-10]. 

**Table 1:** Imaging Studies to be Performed in All Patients with Erdheim-Chester Disease

Imaging Studies to be Performed in All Patients with Erdheim-Chester Disease
CT scan of chest, abdomen, and pelvis
(18F)-fluorodeoxyglucose (FDG) PET of entire body
MRI of the brain with gadolinium
Cardiac MRI

## Conclusions

We present a largely subclinical case of ECD in a 45-year-old man. ECD is a rare disease of unclear origin and may manifest in many ways, though skeletal, aortic, pulmonary, pleural, and renal involvement are common. Thorough imaging and clinical follow-up are essential in these patients as complications can be life-threatening. Definitive treatment is lacking, though recent reports demonstrate the efficacy of BRAF inhibition in certain patient groups.
